# Circulating Autoantibodies Recognizing Immunodominant Epitopes From Human Apolipoprotein B Associate With Cardiometabolic Risk Factors, but Not With Atherosclerotic Disease

**DOI:** 10.3389/fcvm.2022.826729

**Published:** 2022-04-11

**Authors:** Timoteo Marchini, Sara Malchow, Lourdes Caceres, Abed Al Hadi El Rabih, Sophie Hansen, Timothy Mwinyella, Lisa Spiga, Sven Piepenburg, Hauke Horstmann, Tijani Olawale, Xiaowei Li, Lucia Sol Mitre, Mark Colin Gissler, Heiko Bugger, Andreas Zirlik, Timo Heidt, Ingo Hilgendorf, Peter Stachon, Constantin von zur Muehlen, Christoph Bode, Dennis Wolf

**Affiliations:** ^1^Cardiology and Angiology, Medical Center, University of Freiburg, Freiburg im Breisgau, Germany; ^2^Faculty of Medicine, University of Freiburg, Freiburg im Breisgau, Germany; ^3^Facultad de Farmacia y Bioquímica, CONICET, Instituto de Bioquímica y Medicina Molecular, Universidad de Buenos Aires, Buenos Aires, Argentina; ^4^Spemann Graduate School of Biology and Medicine, University of Freiburg, Freiburg im Breisgau, Germany; ^5^Department of Cardiology, University Heart Center Graz, Medical University Graz, Graz, Austria

**Keywords:** atherosclerosis, cardiovascular disease, ApoB, auto-antibodies, immunity

## Abstract

**Rationale:**

Atherosclerosis is a chronic inflammatory disease of large arteries that involves an autoimmune response with autoreactive T cells and auto-antibodies recognizing Apolipoprotein B (ApoB), the core protein of low-density lipoprotein (LDL). Here, we aimed to establish a clinical association between circulating human ApoB auto-antibodies with atherosclerosis and its clinical risk factors using a novel assay to detect auto-antibodies against a pool of highly immunogenic ApoB-peptides.

**Methods and Results:**

To detect polyclonal IgM- and IgG-antibodies recognizing ApoB, we developed a chemiluminescent sandwich ELISA with 30 ApoB peptides selected by an *in silico* assay for a high binding affinity to MHC-II, which cover more than 80% of known MHC-II variants in a Caucasian population. This pre-selection of immunogenic self-peptides accounted for the high variability of human MHC-II, which is fundamental to allow T cell dependent generation of IgG antibodies. We quantified levels of ApoB-autoantibodies in a clinical cohort of 307 patients that underwent coronary angiography. Plasma anti-ApoB IgG and IgM concentrations showed no differences across healthy individuals (*n* = 67), patients with coronary artery disease (*n* = 179), and patients with an acute coronary syndrome (*n* = 61). However, plasma levels of anti-ApoB IgG, which are considered pro-inflammatory, were significantly increased in patients with obesity (*p* = 0.044) and arterial hypertension (*p* < 0.0001). In addition, patients diagnosed with the metabolic syndrome showed significantly elevated Anti-ApoB IgG (*p* = 0.002). Even when normalized for total plasma IgG, anti-ApoB IgG remained highly upregulated in hypertensive patients (*p* < 0.0001). We observed no association with triglycerides, total cholesterol, VLDL, or LDL plasma levels. However, total and normalized anti-ApoB IgG levels negatively correlated with HDL. In contrast, total and normalized anti-ApoB IgM, that have been suggested as anti-inflammatory, were significantly lower in diabetic patients (*p* = 0.012) and in patients with the metabolic syndrome (*p* = 0.005).

**Conclusion:**

Using a novel ELISA method to detect auto-antibodies against ApoB in humans, we show that anti-ApoB IgG associate with cardiovascular risk factors but not with the clinical appearance of atherosclerosis, suggesting that humoral immune responses against ApoB are shaped by cardiovascular risk factors but not disease status itself. This novel tool will be helpful to develop immune-based risk stratification for clinical atherosclerosis in the future.

## Introduction

Cardiovascular disease (CVD) is the leading cause of mortality worldwide (1) and most frequently caused by atherosclerosis, a chronic inflammatory disease characterized by lipid accumulation in the subendothelial space of middle- to large-sized arteries that form vessel-obstructing atherosclerotic plaques (2). The spontaneous rupture of atherosclerotic plaques may ultimately cause an occlusive arterial thrombi that restricts blood flow and precipitates myocardial infarction (MI) and stroke (3). Atherosclerotic lesions develop in arteries with high shear stress, turbulent blood flow, and endothelial dysfunction (4). This process is promoted by traditional cardiovascular risk factors, such as smoking, hypertension, obesity, diabetes, and environmental stressors (5). In atherosclerotic arteries, low-density lipoprotein (LDL) cholesterol particles from the blood circulation are deposited in the subendothelial space and modified by oxidative processes (6). Oxidized LDL (oxLDL) is taken up by tissue-resident macrophages and initiates a pro-inflammatory response that drives disease progression (7). Levels of plasma cholesterol, LDL, and apolipoproteins including Apolipoprotein B (ApoB), the protein backbone of LDL, correlate with clinical atherosclerosis (8). LDL lowering strategies by pharmacological inhibition of the HMG-CoA reductase are recommended in the primary and secondary prevention of atherosclerotic disease (9). However, a relevant inflammatory risk remains even if LDL levels are within the current target ranges (10). Multiple discordance analysis has demonstrated that ApoB has a stronger association with cardiovascular risk than other plasma lipoproteins (11, 12).

Increasing clinical and experimental evidence has highlighted the contribution of an ongoing autoimmune response in atherosclerosis that involves T cells and B cell derived autoantibodies (13). Both CD4^+^ and CD8^+^ T cells accumulate in mouse and human atherosclerotic lesions (14). In *Apoe*^–/–^ mice, a population of CD4^+^ T cells recognizes ApoB *self*-peptides presented in an MHC-II dependent fashion (15, 16). Autoreactive T cells expand within the atherosclerotic plaque of mice and humans (17, 18). In addition to an autoreactive T cell response, autoantibodies recognizing LDL, oxLDL, and ApoB have been detected in the plasma of mice and humans with atherosclerosis (6, 19). Naturally occurring IgM antibodies directed against oxLDL epitopes are generally considered atheroprotective (20). The role of IgG in the pathogenesis of atherosclerosis is still under debate as IgGs recognizing oxLDL have been shown to aggravate (21) or protect (22) from atherosclerosis in mice. Likewise, studies in humans have suggested that anti-ApoB autoantibodies are atheroprotective (23–27) or pro-atherogenic (28). Although several studies have tested the association of IgG and IgM autoantibodies in a clinical setting [reviewed here (29)], most studies interrogated humoral responses to single self-peptides from ApoB, thus, not allowing to assess the net effect of a polyclonal antibody response against the complex antigen ApoB. In addition, previous studies have not accounted for the genetic diversity of MHC-II allele expression in humans that may affect the generation of autoantibodies against specific ApoB-peptides. Collectively, the association of anti-ApoB autoantibodies and cardiovascular risk factors remains only poorly defined. Here, we aim to circumvent these limitations by the quantification of autoantibodies recognizing a pool of ApoB derived-peptides with proven MHC-II affinity in a single-centre study with 307 participants.

## Materials and Methods

**FIGURE 1:**
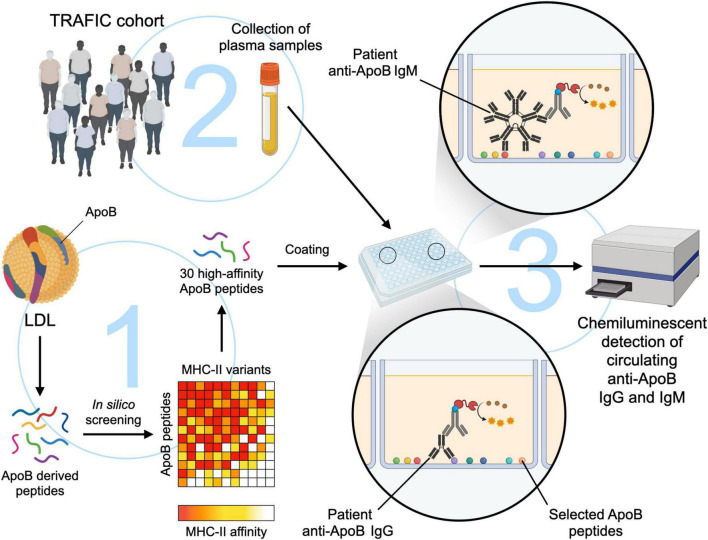
Study workflow for the development of a novel ELISA to detect anti-ApoB IgG- and IgM- plasma levels. (1) ApoB-derived peptides with a length of 15 amino acids were screened *in silico* for their binding affinity to MHC-II variants (18). A pool of 30 peptides that showed a high affinity to MHC-II in direct *in vitro* affinity measurements were used to coat a microplate at 5 μg/mL. (2) A total of 307 plasma samples from the TRAFIC cohort were analyzed by an *in-house* chemiluminescent ELISA (3) to detect circulating anti-ApoB IgG and IgM auto-antibodies. The figure was generated with schematics from Biorender.com.

The study workflow is summarized in Figure 1. An Expanded Methods Section is available in the online-only Data Supplement.

### Patient Cohort

Human plasma samples were obtained from the Tumor Necrosis Factor Receptor Associated Factors in Cardiovascular Risk Study (TRAFIC) at the University Heart Center Freiburg, University of Freiburg, Germany, that underwent coronary angiography (30). We analyzed plasma samples from 67 patients without relevant coronary atherosclerosis (healthy), 179 patients with coronary artery disease (CAD), and 61 patients presenting with a coronary syndrome (ACS). CAD was defined as the presence of at least one coronary stenosis >50% in coronary angiography. ACS was defined as the presence of unstable angina, non-ST-segment elevation myocardial infarction (NSTEMI), or ST-segment elevation myocardial infarction (STEMI). Type 2 diabetes mellitus (T2DM) and hypertension (HTN) were either defined by pre-documented disease or HbA1c > 6.5% (T2DM) or blood pressure >140/90 mmHg (hypertension) at the time of presentation. Obesity was defined by a body mass index (BMI) >35. Metabolic Syndrome (MS) was defined by the presence of at least 3 metabolic conditions (obesity, arterial hypertension, hypercholesterolemia, T2DM, and hypertriglyceridemia) according to a modified definition of the IDF (31). Standard laboratory assays were performed by the local clinical laboratory at the University Hospital Freiburg, Germany. All procedures were carried out according to institutional guidelines (approval number 75/06 and 22-1046 by the local ethics committee, University Hospital of Freiburg, Germany) and the declaration of Helsinki.

### Total Plasma IgG and IgM Levels

Total plasma IgG and IgM levels were quantified using a Flex Set Cytometric Bead Array (BD Biosciences, San José, CA, United States) according to the manufacturer’s protocol. Briefly, human plasma samples were diluted in assay buffer at 1:200,000 for total IgG and 1:4,000 for total IgM and incubated for 1 h with the supplied beads in a V-Bottom 96 well plate at room temperature. Subsequently, samples were washed and incubated with a PE-detection reagent for 2 h at room temperature. Samples were acquired on a FACS Canto II flow cytometer (BD Biosciences) and analyzed with FlowJo software v10 (Tree Star, Ashland, United States). Absolute concentrations (in μg/mL) were calculated using a concentration curve with purified IgG and IgM.

### Plasma ApoB_100_ Levels

Patient plasma ApoB_100_ levels were measured using a Human ApoB Quantikine ELISA Kit (R&D systems, Minneapolis, MN, United States) according to the manufacturer’s protocol. Samples were denatured, diluted at 1:2,000, and incubated with the capture and detection antibodies for 1 h at room temperature. After washing, TMB development solution was added and incubated for 15 min. Sample optic density (OD) was determined at 450 nm using a SpectraMax M2 Microplate Reader (Molecular Devices, San Jose, CA, United States).

### Development of an ELISA to Detect Circulating Anti-ApoB IgG and IgM

30 ApoB_100_ peptides with a high affinity to several human MHC-II variants were chosen by *in silico* screening and direct *in vitro* affinity measurements (18). Peptides were manufactured by Peptide Specialty Laboratories GmbH (Heidelberg, Germany), diluted in DMSO at 20 mg/mL, pooled, and frozen at −80°C until used. To detect plasma levels of anti-ApoB auto-antibodies by ELISA, the peptide pool was used to coat white U-bottom microplates (BrandTech Scientific, Essex, CT, United States) at 5 μg/mL. A standard curve was prepared by coating the plate with increasing concentrations of pure human IgG or IgM (Jackson ImmunoResearch, Cambridgeshire, United Kingdom). After overnight incubation at 4°C, plates were washed and blocked with 1% BSA. After 1 h at room temperature, plates were washed and 50 μL of diluted plasma samples (1:50) were added to the wells in duplicates. After 90 min at room temperature, plates were washed and incubated with a horseradish peroxidase (HRP) conjugated anti-human IgG or IgM antibody (1:20,000, Jackson ImmunoResearch). After 1 h at room temperature, plates were washed and incubated with the SuperSignal ELISA Femto Substrate (Thermo Fisher Scientific, Waltham, MA, United States). Luminescence was measured 1 min after addition of the substrate in an Infinite 200 PRO microplate reader (Tecan, Switzerland). Patient anti-ApoB IgG or IgM plasma levels were obtained using the corresponding standard curve adjusted to a 5-parameter logistic curve fit, resulting in a dynamic range of 1.16–66.67 ng/mL for the IgG assay and of 0.51–29.63 ng/mL for the IgM assay. Alternatively, background signal in DPBS coated wells (blank) was subtracted from raw RLU values (RLU-blank). As indicated, single ApoB-peptides were used instead of the pool of 30 peptides at the same total peptide concentration.

### Statistics

Statistical analysis was performed using GraphPad Prism software v8.0. As data was not normally distributed, a Mann–Whitney test was used for the comparison of two groups and the Kruskal–Wallis test followed by Dunn’s multiple comparisons for the comparison of more than two groups. Statistical significance was considered at *p* < 0.05. Data are presented as median or as otherwise indicated.

## Results

### Clinical Characteristics of the Patient Cohort

The TRAFIC study population comprises a total of 307 patients that underwent coronary angiography at the University Heart Center, Freiburg. 58.3% of patients were diagnosed with CAD, 19.9% presented with an ACS. Expectedly, we detected an increased frequency of T2DM, smoking, arterial hypertension, and dyslipidaemia among patients with CAD/ACS compared to subjects absent of CAD. Patients with ACS had higher concentrations of circulating C-Reactive Protein (CRP). Body Mass Index (BMI), the prevalence of obesity, and levels of serum glucose, HbA1c, and triglycerides were not significantly changed between the groups. Clinical characteristics are presented in Table 1.

**TABLE 1:** Clinical characteristics of the TRAFIC study population.

	no CAD (*n* = 67)	CAD (*n* = 179)	ACS (*n* = 61)
BMI (kg/m^2^)	27.6 ± 4.5	27.6 ± 4.4	28.3 ± 4.3
Age (years)	62.5 ± 8.7	65.7 ± 8.3*	64.3 ± 8.9
CRP (mg/L)	3.2 ± 10.6	3.6 ± 8.6^###^	17.6 ± 40.7^§§§^
Creatinine (mg/dL)	0.9 ± 0.2	1.1 ± 0.7**	1.0 ± 0.3^§§^
Prior MI (%)	0.0 (0)	40.2 (72)*** ^##^	20.0 (12)^§§§^
Sex (% male)	64.2 (43)	84.4 (151)**	77.1 (47)
Diabetes Mellitus Type 2 (%)	9.0 (6)	25.1 (45)**	27.9 (17)^§^
Smoking (%)	32.8 (22)	52.0 (93)^*,##^	72.1 (44)^§§§^
Serum Glucose (mg/dL)	111 ± 21	120 ± 39	121 ± 32
HbA1c (%)	5.9 ± 0.6	6.2 ± 0.8	6.4 ± 0.9
Leukocytes (x10^6^/mL)	6.8 ± 1.8	7.0 ± 2.1^###^	8.8 ± 3.1^§§§^
Total Cholesterol (mg/dL)	199 ± 42	181 ± 42*	195 ± 51
Triglycerides (mg/dL)	127 ± 69	162 ± 99	166 ± 107
LDL (mg/dL)	116 ± 31	99 ± 34*	99 ± 43
VLDL (mg/dL)	30.0 ± 15.2	34.9 ± 17.7	39.7 ± 19.9^§^
HDL (mg/dL)	53.7 ± 16.1	45.3 ± 13.3*	47.7 ± 18.5
Arterial Hypertension (%)	55.2 (37)	74.3 (133)**	77.1 (47)^§^
Hypercholesterolemia (%)	19.4 (13)	51.4 (92)***	47.5 (29)^§§§^
Obesity (%)	22.4 (15)	25.7 (46)	27.9 (17)
Metabolic Syndrome (%)	32.8 (22)	52.5 (94)**	57.4 (35)^§§^

*Categorical variables are expressed in percentages within the groups (total number depicted in brackets), continuous variables as mean ± SD. Statistical significance was tested using Kruskal–Wallis test followed by multiple comparisons for continuous variables, using Fisher’s exact test for categorical variables. MI, myocardial infarction.*

*Indicates significance between no CAD and CAD (*p < 0.05, **p < 0.01, ***p < 0.001).

*^§^indicates significance between no CAD and ACS (^§^p < 0.05, ^§§^p < 0.01, ^§§§^p < 0.001).*

*^#^indicates significance between CAD and ACS (^#^p < 0.05, ^##^p < 0.01, ^###^p < 0.001).*

In a subgroup analysis, patients with T2DM showed increased BMI, serum glucose, and HbA1c (Supplementary Table 1). Among smokers, a higher frequency of males and dyslipidaemia was detected (Supplementary Table 2) as well as higher circulating leukocyte numbers. In patients with arterial hypertension, obesity, and MS, we detected increased BMI, serum glucose, HbA1c, as well as a higher prevalence of hypertriglyceridemia and dyslipidaemia (Supplementary Tables 3–5).

### Total Plasma IgG and IgM Levels Are Not Regulated in Patients With Clinical Atherosclerosis

**FIGURE 2:**
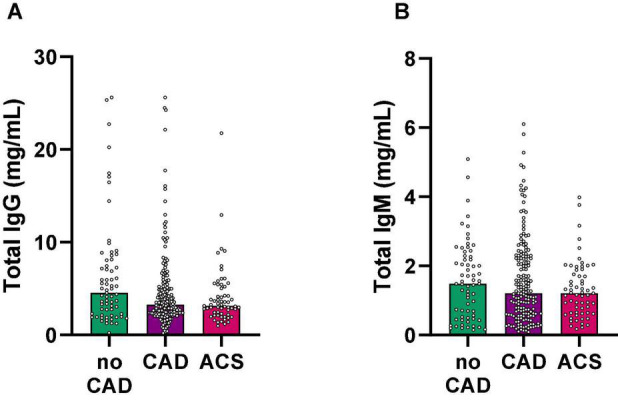
Total IgG and IgM plasma levels do not associate with coronary atherosclerosis in coronary angiography. **(A)** Total IgG and **(B)** IgM levels were quantified in plasma samples using a Flex Set Cytometric Bead Array (BD Biosciences) and grouped according to the presence of coronary atherosclerosis in coronary angiography. CAD, coronary artery disease; ACS, acute coronary syndrome.

To investigate differences in ApoB-specific autoantibodies, we first aimed to clarify total IgG and IgM levels. Importantly, we detected no significant differences in total IgG or IgM plasma levels among healthy (no CAD), CAD, and ACS patients (Figure 2). Quartile distribution of total IgG and IgM plasma levels according to patient diagnosis is provided in Supplementary Table 6. Our data indicate that total concentrations of immunoglobulins do not significantly differ between patients with and without clinically relevant coronary atherosclerosis.

### Anti-ApoB IgG Plasma Levels Are Increased in Patients at High Cardiovascular Risk

**FIGURE 3:**
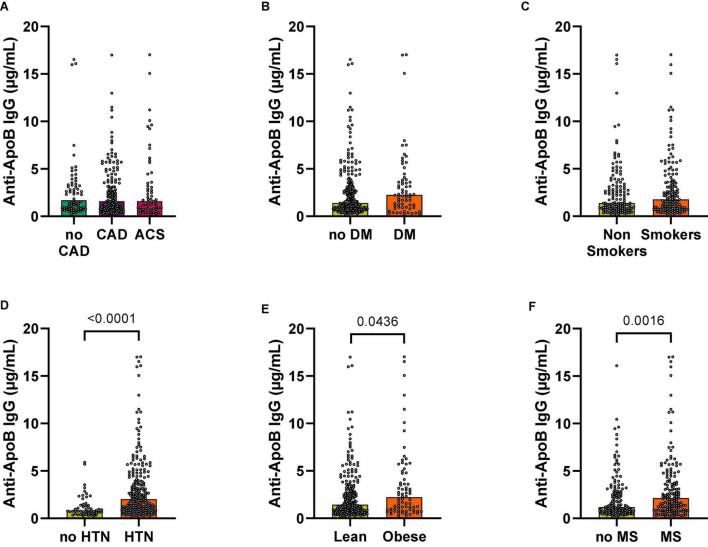
Anti-ApoB IgG plasma levels are increased in patients with hypertension and obesity. Anti-ApoB IgG plasma levels were quantified by ELISA and grouped according to **(A)** patient diagnosis or the presence of cardiometabolic risk factors **(B–F)**. CAD, coronary artery disease; ACS, acute coronary syndrome; DM, diabetes mellitus; HTN, hypertension; MS, metabolic syndrome.

We next tested the association of ApoB-specific antibodies and the clinical appearance of coronary atherosclerosis. Surprisingly, the concentration of specific IgGs binding to the ApoB peptide pool (anti-ApoB IgG) was not changed between patients without CAD (no CAD) and those with CAD and presenting with an ACS (Figure 3A). This was consistent even after normalization for total IgG levels in each participant (Supplementary Figure 1A). Because angiographically-defined coronary atherosclerosis may not be the major determinant of autoantibody generation, we next grouped the study participants according to the presence of cardiometabolic risk factors: While anti-ApoB IgG showed no significant regulation in patients with T2DM (Figure 3B) and smoking status (Figure 3C), plasma anti-ApoB IgG was significantly increased in patients with arterial hypertension [1.27 ± 0.17 μg/mL (no hypertension) vs. 3.42 ± 0.30 μg/mL (hypertension)], obesity [2.81 ± 0.29 μg/mL (lean) vs. 3.72 ± 0.53 μg/mL (obese)], and the Metabolic Syndrome [2.81 ± 0.29 μg/mL (no MS) vs. 3.72 ± 0.53 μg/mL (MS)], suggesting a positive association of these cardiometabolic risk factors and anti-ApoB IgG levels (Figures 3D–F). After normalizing for total IgG levels, anti-ApoB IgG levels remained highly upregulated in hypertensive patients (Supplementary Figure 1D). We detected no association of anti-ApoB IgG with plasma CRP levels (Supplementary Figure 2). These data indicate that anti-ApoB IgG antibody levels are specifically increased in patients at high cardiometabolic risk, particularly in those with arterial hypertension.

Because total anti-ApoB IgG levels reflect a mix of several monoclonal IgG-antibody clones that can bind to one of the 30 ApoB-peptides, we next interrogated whether the increase of total anti-ApoB IgGs was driven by a small number of predominant ApoB-peptides only. We therefore selected a sub-group of patients with the 10 highest individual anti-ApoB IgG levels with clinical obesity and hypertension (“high” CVD risk) and with the 10 lowest individual anti-ApoB IgG levels without clinical obesity and hypertension (“low” CVD risk). We observed relevant anti-ApoB IgG titers against all ApoB-peptides (Supplementary Figure 3A) with a low standard deviation (41.2% of mean values) and an overall 5.8-fold difference between the peptide with the lowest (peptide 19) and highest (peptide 24) average anti-ApoB IgG signals. While we detected varying patterns of high anti-ApoB IgG titers against a small or larger number of ApoB-peptides within one patient (Supplementary Figure 3B), anti-ApoB IgG titers against 19 of the 30 ApoB-peptides were higher or showed a strong tendency to be increased in patients with a high CVD risk compared with those with a low CVD risk (Supplementary Figure 4). These data indicate that although patients may preferably express antibodies against specific peptides, the observed increase of anti-ApoB IgG antibodies in obese and hypertensive patients is caused by a broad range of ApoB self-peptides.

### Anti-ApoB IgG Plasma Levels Are Associated With Lower HDL and Apolipoprotein B Levels

**FIGURE 4:**
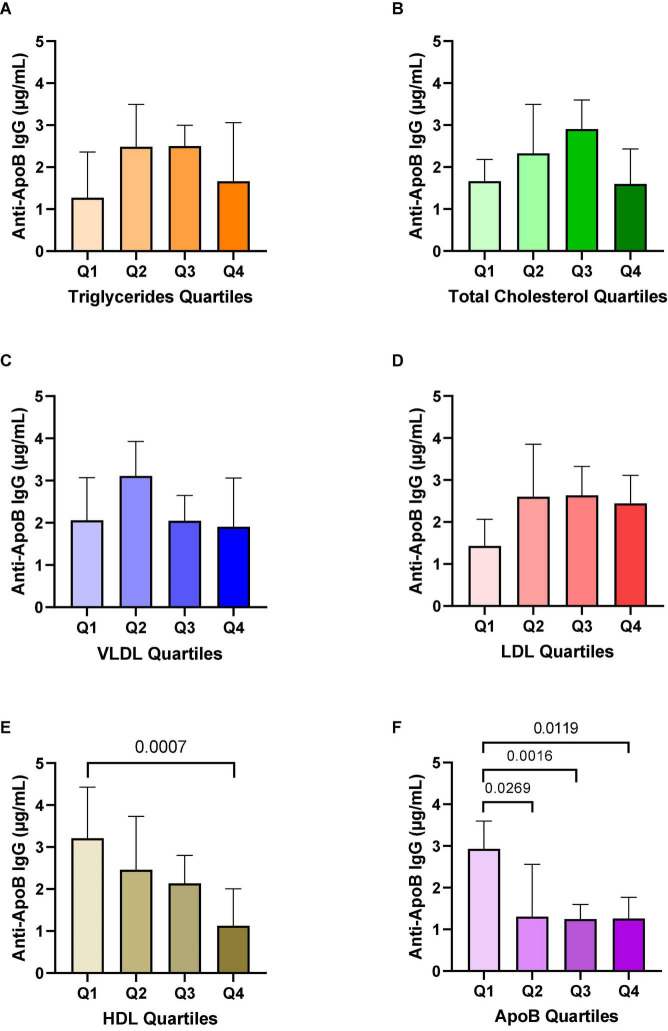
Associations of anti-ApoB IgG plasma levels with triglycerides, cholesterol, and apolipoproteins. Anti-ApoB IgG plasma levels were quantified by ELISA and divided into quartiles (Q) of patient plasma levels of **(A)** triglycerides, **(B)** total cholesterol, **(C)** VLDL, **(D)** LDL, **(E)** HDL, and **(F)** ApoB. VLDL, very low-density lipoprotein; LDL, low-density lipoprotein; HDL, high-density lipoprotein. Data are presented as median ± 95%CI.

Since the secretion of autoantibodies is instructed by the presence of the cognate autoantigen, we speculated that levels of anti-ApoB IgG would associate with the plasma concentration of the ApoB-containing apolipoproteins LDL and very low-density lipoprotein (VLDL). Expectedly, we did not find an association to non-ApoB containing triglycerides (Figure 4A). However, we did not detect a relevant difference of anti-ApoB IgG levels to quartiles of cholesterol (Figure 4B), VLDL (Figure 4C), and LDL (Figure 4D). Interestingly, we detected an inverse correlation of anti-ApoB IgG concentrations and high-density lipoprotein (HDL, Figure 4E). HDL is known to negatively correlate to CVD but does not carry ApoB itself (32). Furthermore, we detected a negative association of anti-ApoB IgG levels with total ApoB concentrations in plasma (Figure 4F). These effects remained significant after normalizing to total IgG levels (Supplementary Figure 5) and support a clinically relevant association between ApoB-autoantibodies and apolipoprotein metabolism.

### Anti-ApoB IgM Plasma Levels Are Decreased in Patients With a High Cardiovascular Risk

**FIGURE 5:**
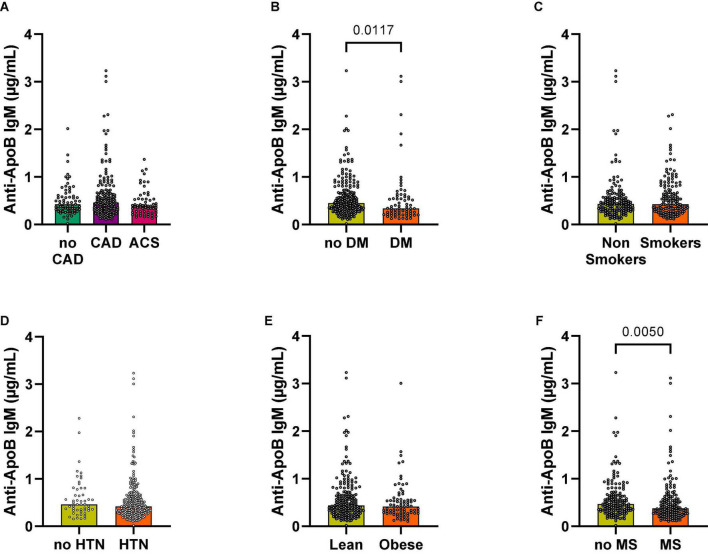
Associations of anti-ApoB IgM plasma levels with cardiovascular risk factors. Anti-ApoB IgM plasma levels were quantified by ELISA and grouped according to **(A)** patient diagnosis or **(B–F)** cardiometabolic risk factors. CAD, coronary artery disease; ACS, acute coronary syndrome; DM, diabetes mellitus; HTN, hypertension; MS, metabolic syndrome.

Anti-LDL IgM antibodies mostly recognize oxLDL epitopes with oxidized phosphocholine and malondialdehyde (MDA)−modified amino groups. Anti-LDL IgM antibodies have been detected in mice and humans (33) and seem to have atheroprotective effects (20). To clarify the relation of IgM autoantibodies recognizing unmodified ApoB-peptides, we quantified IgM antibodies in parallel to anti-ApoB IgG antibodies. Both, total IgM plasma levels (Figure 2B) and specific anti-ApoB IgM levels, showed no relevant differences among patients without CAD (no CAD), CAD, and these presenting with an ACS (Figure 5A). We obtained similar findings after normalizing for total IgM levels (Supplementary Figure 6). We detected that anti-ApoB IgM plasma levels were significantly decreased in patients with T2DM (Figure 5B) and the MS (Figure 5F) but were not modulated by smoking status (Figure 5C), arterial hypertension (HTN, Figure 5D), or obesity (Figure 5E). The negative association with T2DM and MS remained significant after controlling for total IgM levels (Supplementary Figure 6). In contrast to anti-ApoB IgG antibodies, anti-ApoB IgM plasma levels did not associate with the lipoprotein profile (Supplementary Figures 7, 8) but where significantly higher in patients within the highest ApoB quartile normalized for total IgM (Supplementary Figure 8F). Collectively, these data suggest an opposing regulation of anti-ApoB IgM and IgG by cardiometabolic risk factors and ApoB in patients with CVD.

## Discussion

This study interrogated the relationship between IgM and IgG anti-ApoB autoantibodies in patients with coronary artery disease (CAD) and traditional cardiometabolic risk factors. We found – partially in contrast to existing evidence (23–25, 27) – that antibodies binding to ApoB-peptides do not primarily associate with clinically relevant coronary atherosclerotic disease but with cardiometabolic risk factors. We observed the strongest regulation in patients with arterial hypertension. Along with the concept that IgM immunity is atheroprotective, while IgG antibodies recognizing LDL, ApoB, or peptides from ApoB are pathogenic mediators (20), we found an opposing regulation of IgG and IgM. In addition, our study is the first to demonstrate a positive correlation between obesity and anti-ApoB IgG levels, while previous studies have proposed a negative association with metabolic risk factors (23, 25, 26). These findings emphasize the relation of metabolism and atherosclerosis-relevant autoimmunity, particularly in the light of the emerging field of immunometabolism (34) and novel anti-diabetic and anti-obesity therapies that improve the outcome of atherosclerotic disease (35).

Autoimmunity in atherosclerosis is a delicate balance of protective and pathogenic events that has been best established in T cellular immunity against ApoB (13). Growing evidence has demonstrated the existence of autoreactive CD4^+^ T-helper cells in the blood of mice and humans (15, 18). These autoreactive T cells mostly express surface markers, cytokines, and transcriptomes reminiscent of immune-suppressive T-regulatory cells (16, 18). In mouse models, adoptive transfers of these cells protect from atherosclerosis. In the later stages, however, ApoB-specific T cells transform into their pathogenic counterparts that are closer to pathogenic T_*H*_1-polarized cells than T_regs_ (18). In addition, a subpopulation of T cells in the atherosclerotic plaque and in lymph nodes differentiates into T follicular helper cells (T_FH_) that aid B cells in maturation, IgG class switch, and their differentiation in plasma cells, which express anti-ApoB autoantibodies. A lack of T_FH_ protects from atherosclerosis (36). *Per se*, the generation of ApoB-autoantibodies represents an event of late-stage atherosclerosis, in which pathogenic T cell phenotypes and effector functions dominate protective limbs of autoimmunity (37). In line, it has been postulated that IgG-mediated LDL complex formation and opsonization may enhance lipid uptake into plaque phagocytes and promote atherosclerosis (21, 38), Conversely, vaccination with ApoB-peptides protects from atherosclerosis but it is unclear whether this is an effect of vaccination-induced T_reg_-like CD4^+^ T-helper cells or of a humoral response involving ApoB-autoantibodies (39). Notably, anti-ApoB antibodies could help clearing LDL particles from the circulation (40) or block its uptake by macrophages and prevent atherosclerosis (20, 22, 41–43). Reports on vaccination-induced mechanisms have been controversial and there is still a considerable lack of knowledge. Likewise, clinical findings remain hard to interpret. For instance, in most studies non-modified ApoB-IgGs inversely correlate with clinical disease (23, 24, 26–28), while antibodies to modified LDL positively correlates with disease in one study allowing a direct comparison (28). Associations with cardiometabolic risk factors in these studies remain highly heterogenous. The largest clinical association study with more than 3,500 participants highlighted a fine-tuned relationship to sex, age, ethnicity and partially opposing effects for ApoB and LDL-specific antibodies (28). Apart from these associative studies, one clinical trial tested the direct transfer of a monoclonal antibody recognizing oxidized LDL in patients with CAD but failed to reduce plaque inflammation in FDG-PET imaging (44).

There are several important differences among the available studies that may explain such heterogeneity: First, it is important to note that the immune response against complex LDL particles and single ApoB-peptides may substantially differ. This is exemplified by the observation that germ-line encoded, naturally occurring IgM recognize modified lipids, which share structural homologies with evolutionary conserved foreign patterns, such as from bacteria or viruses (20). Therefore, lipid-specific (oxidations-specific) and T cell dependent, anti-peptide antibodies may have fundamentally different roles in host defense, inflammation, and autoimmunity. Second, the fact that antibodies recognize peptides that are located in the inner core of LDL particles or peptides that are exposed to the hydrated surface and therefore accessible to circulating antibodies may relevantly impact on their biological function. Vaccination studies have demonstrated antibody-responses to a wide range of peptides independent of their location within LDL (39). While antibodies may represent a biomarker of vaccination, they may not necessarily exhibit biological function, in particular when the respective peptide is not accessible to circulating antibodies because of its location inside LDL particles. During the intracellular processing of LDL-containing peptides by APCs, however, antibodies without a biological function may be generated as bystander-response and used as biomarker of the specific immune risk. Third, to quantify antibodies with a specificity against a single peptide in a mixed population is potentially under-estimating potential effects. Since the generation of these antibodies depends on T_FH_ that provide help to differentiating B cells it is more likely that a peptide with a high-affinity for MHC-II is presented by respective B cells to a T cell with a high-affinity TCR for this MHC-II:peptide interaction. Because several thousands of different MHC-II alleles are expressed in the human population, it is unlikely that a single peptide would exhibit high-affinities across all MHC-II variants. Notably, MHC-II typing has not been performed in the available ApoB antibody trials so far. In our study, we used a pool of 30 ApoB-peptides that have been carefully selected to represent sufficient binding affinities across a wide range of MHC-II alleles (18). We suggest that the multi-peptide design of our study is more representative and potentially biologically relevant than single-peptide IgG targets. We detected a clear predominance of antibodies against some peptides in individual patients. This pattern showed a considerable variation across patients, which supports the idea that individual MHC-II variants respond differently to certain ApoB-peptides. It is also important to note that even IgG-antibodies recognizing only one ApoB-peptide may consist of a polyclonal repertoire of different IgG-antibody clones with varying affinities toward the same peptide. Our findings therefore suggest that reported clinical associations may be under-estimated in available studies testing auto-antibodies against one peptide only. On the contrary, studies quantifying autoantibodies to the entire ApoB protein are at the risk of lower specificity. Interestingly, our study revealed that the peptide with highest individual ApoB-IgG signal (peptide 24) was not regulated between patients with a low and high risk for CVD. Therefore, a pool of selected peptides should be broad enough to account for naturally occurring MHC-II alleles and small enough to allow tracking of single peptide-specificities and to minimize unspecific peptide-specific IgGs that are not regulated in disease.

Our study raises some important questions: For instance, it is intriguing to ask whether hypertension remains independently associated with ApoB IgG levels and how arterial hypertension may drive IgG generation. Associations with arterial hypertension have (26, 28) and have not been demonstrated before (25). It will also be important to clarify the relation between autoantigens, humoral, and cellular responses. While we have previously shown the existence of a T cell population circulating in human blood that recognizes the same pool of ApoB peptides, it will be necessary to clarify the association of autoantibodies and antigen-specific T cells on a single peptide basis. Particularly, it would be interesting to interrogate whether T_FH_ with the same peptide specificity would exist and associate with antibodies recognizing the same peptide. Although the inverse correlation of ApoB concentrations and ApoB-autoantibodies seems to be counter-intuitive, it is plausible to speculate whether the chronic stimulation of humoral and cellular limbs of autoimmunity would result in immune exhaustion with a subsequent loss of ApoB-specific plasma cells and a decline of respective auto-antibodies over time. In this case, an inverse correlation of disease progression and antibody concentrations should not be interpreted as causal. Eventually, the understanding of the precise interplay of humoral and cellular autoimmune mechanisms will allow the development of novel humoral and cellular risk stratification tools to identify CVD patients at high immune risk.

## Data Availability Statement

The raw data supporting the conclusions of this article will be made available by the authors, without undue reservation.

## Ethics Statement

The studies involving human participants were reviewed and approved by the Ethics Committee, University Hospital of Freiburg, Germany (Approval numbers 75/06 and 22-1046). The patients/participants provided their written informed consent to participate in this study.

## Author Contributions

All authors listed made a substantial, direct, and intellectual contribution to this work, and approved it for publication.

## Conflict of Interest

The authors declare that the research was conducted in the absence of any commercial or financial relationships that could be construed as a potential conflict of interest.

## Publisher’s Note

All claims expressed in this article are solely those of the authors and do not necessarily represent those of their affiliated organizations, or those of the publisher, the editors and the reviewers. Any product that may be evaluated in this article, or claim that may be made by its manufacturer, is not guaranteed or endorsed by the publisher.
