# Supplementary material for: Circulating Autoantibodies Recognizing Immunodominant Epitopes From Human Apolipoprotein B Associate With Cardiometabolic Risk Factors, but Not With Atherosclerotic Disease
Source: Front Cardiovasc Med. 2022 Apr 11;9:826729. doi: 10.3389/fcvm.2022.826729 (PMC9035541; doi:10.3389/fcvm.2022.826729)
Supplement: Supplementary file 1 [file Data_Sheet_1.docx]

**Data Supplement**

**Circulating autoantibodies recognizing immunodominant epitopes from human Apolipoprotein B (ApoB) associate with cardiometabolic risk factors, but not with atherosclerotic disease**

Timoteo Marchini, Sara Malchow, Lourdes Caceres, Abed Al Hadi El Rabih, Sophie Hansen, Timothy Mwinyella, Lisa Spiga, Sven Piepenburg, Hauke Horstmann, Tijani Olawale, Xiaowei Li, Lucia Sol Mitre, Mark Colin Gissler, Heiko Bugger, Andreas Zirlik, Timo Heidt, Ingo Hilgendorf, Peter Stachon, Constantin von zur Muhlen, Christoph Bode, and Dennis Wolf

**Content:**

- Expanded Methods Section
- Supplementary Tables (6)
- Supplementary Figures (8)
- Supplementary References (1)

**Expanded Methods Section**

*ELISA to detect circulating anti-ApoB IgG and IgM*

30 peptides of the entire ApoB_100_ sequence with a length of 15 amino acids were selected by an *in-silico* screening based on high binding affinity to MHC-II variants and direct *in vitro* affinity measurements as reported previously (1). Peptides were generated by Peptide Specialty Laboratories (Heidelberg, Germany), resuspended in DMSO at 20 mg/mL, and frozen at -80 °C. Peptides were pooled by mixing 30μL of the 30 single peptides. The peptide pool was aliquoted and frozen at -80 °C. The peptide pools were thawed up immediately before starting the coating step in the ELISA protocol. To detect plasma anti-ApoB auto-antibodies by ELISA, the peptide pool was used to coat white U-bottom microplates (BrandTech Scientific, Essex, CT, US) at 5 µg/mL. A standard curve was prepared by coating additional wells with increasing concentrations of pure human IgG or IgM (Jackson ImmunoResearch, Cambridgeshire, UK) instead of the peptide pool. For coating, both the standards and the peptide pool were diluted in DPBS. After overnight incubation at 4 °C, the plate was washed three times with DPBS and blocked with 1% BSA in DPBS. After 1 h at room temperature, the plate was washed three times with DPBS and 50 µL of plasma samples diluted 1:50 in 1% TBS and 0.1% Tween20 was added in duplicates to the wells coated with the peptide pool. After 1.5 h at room temperature, the plate was washed three times with TBS-Tween20 and incubated with a horseradish peroxidase (HRP) conjugated anti-human IgG or IgM antibody (Jackson ImmunoResearch) diluted 1:20,000 in 1% BSA-TBS. After 1 h at room temperature, the wells were washed three times with TBS-Tween20 and four times with distilled H_2_O. Subsequently, the SuperSignal ELISA Femto Maximum Sensitivity Substrate (Thermo Fisher Scientific, Waltham, MA, US) prepared as a 1:1 mixture of the two supplied reagents was added. Luminescence was measured as relative luminescence units (RLU) 1 min after adding the substrate in an Infinite 200 PRO microplate reader (Tecan, Switzerland). Patient anti-ApoB IgG and IgM plasma levels were obtained using the corresponding standard curve adjusted to a 5-parameter logistic curve fit, resulting in a dynamic range of 1.16 ng/mL to 66.67 ng/mL for the IgG assay and of 0.51 ng/mL to 29.63 ng/mL for the IgM assay. Alternatively, background signal in DPBS coated wells (blank) was subtracted from raw RLU values (RLU-blank). As indicated, single ApoB-peptides were used instead of the pool of 30 peptides at the same total peptide concentration.

**Supplementary Tables**

**Supplementary Table S1: Clinical characteristics of patients with or without type 2 diabetes mellitus (DM).**

|  | **no DM (n=238)** | **DM (n=68)** |
| --- | --- | --- |
| **BMI (kg/m^2^)** | 27.3 ± 4.3 | 29.1 ± 4.4 ** |
| **Age (years)** | 63.9 ± 8.7 | 67.5 ± 7.6 ** |
| **CRP (mg/L)** | 5.7 ± 19.1 | 9.1 ± 26.8 |
| **Creatinine (mg/dL)** | 1.0 ± 0.6 | 1.1 ± 0.4 |
| **Prior MI (%)** | 26.5 (63) | 30.9 (21) |
| **Sex (% male)** | 78.6 (187) | 77.9 (53) |
| **Smoking (%)** | 51.7 (123) | 52.9 (36) |
| **Serum Glucose (mg/dL)** | 110 ± 22 | 147 ± 50 *** |
| **HbA1c (%)** | 5.9 ± 0.5 | 7.1 ± 1.0 *** |
| **Leukocytes (x10^6^/mL)** | 7.2 ± 2.3 | 7.6 ± 2.6 |
| **Total Cholesterol (mg/dL)** | 190 ± 44 | 176 ± 44 * |
| **Triglycerides (mg/dL)** | 150 ± 85 | 177 ± 125 |
| **LDL (mg/dL)** | 105 ± 35 | 91.3 ± 35.6 * |
| **VLDL (mg/dL)** | 33.6 ± 14.3 | 39.1 ± 25.3 |
| **HDL (mg/dL)** | 48.6 ± 15.7 | 43.0 ± 12.4 * |
| **ApoB (µg/mL)** | 208 ± 157 | 195 ± 134 |
| **Arterial Hypertension (%)** | 79.8 (190) | 92.6 (63) * |
| **Hypercholesterolemia (%)** | 41.6 (99) | 51.5 (35) |
| **Obesity (%)** | 21.4 (51) | 38.2 (26) ** |
| **Metabolic Syndrome (%)** | 39.1 (93) | 85.3 (58) *** |
| **Total IgG (mg/mL)** | 5.0 ± 4.7 | 4.6 ± 4.0 |
| **Total IgM (mg/mL)** | 1.5 ± 1.2 | 1.5 ± 1.1 |
| Categorical variables are expressed as percentages within the groups (total number depicted in brackets), continuous variables as mean ± SD. Statistical significance was tested using Mann-Whitney test for continuous variables, using Chi-square test for diagnosis and Fisher's exact test for categorical variables. * indicates significance between no DM and DM (*p<0.05, **p<0.01, ***p<0.001). | | |

**Supplementary Table S2: Clinical characteristics of smokers and non-smokers.**

|  | **Non-Smokers (n=144)** | **Smokers (n=159)** |
| --- | --- | --- |
| **BMI (kg/m^2^)** | 27.2 ± 3.7 | 28.2 ± 4.9 |
| **Age (years)** | 67.6 ± 7.4 | 62.0 ± 8.6 *** |
| **CRP (mg/L)** | 6.6 ± 22.5 | 6.2 ± 20.0 * |
| **Creatinine (mg/dL)** | 1.1 ± 0.8 | 1.0 ± 0.3 |
| **Prior MI (%)** | 25.0 (36) | 29.6 (47) |
| **Sex (% male)** | 68.8 (99) | 87.4 (139) *** |
| **Diabetes Mellitus Type 2 (%)** | 21.5 (31) | 22.6 (36) |
| **Serum Glucose (mg/dL)** | 120 ± 33 | 116 ± 35 |
| **HbA1c (%)** | 6.2 ± 0.7 | 6.3 ± 0.9 |
| **Leukocytes (x10^6^/mL)** | 6.8 ± 2.1 | 7.8 ± 2.5 *** |
| **Total Cholesterol (mg/dL)** | 186 ± 47 | 187 ± 40 |
| **Triglycerides (mg/dL)** | 145 ± 87 | 168 ± 104 * |
| **LDL (mg/dL)** | 99.4 ± 36.7 | 103 ± 35 |
| **VLDL (mg/dL)** | 32.1 ± 15.3 | 37.3 ± 19.6 * |
| **HDL (mg/dL)** | 50.2 ± 16.4 | 44.6 ± 13.7 * |
| **ApoB (µg/mL)** | 215 ± 152 | 194 ± 153 |
| **Arterial Hypertension (%)** | 84.7 (122) | 81.8 (130) |
| **Hypercholesterolemia (%)** | 38.9 (56) | 49.1 (78) |
| **Obesity (%)** | 21.5 (31) | 28.9 (46) |
| **Metabolic Syndrome (%)** | 47.2 (68) | 51.6 (82) |
| **Total IgG (mg/mL)** | 5.4 ± 4.8 | 4.5 ± 4.2 |
| **Total IgM (mg/mL)** | 1.5 ± 1.1 | 1.5 ± 1.2 |
| Categorical variables are expressed as percentages within the groups (total number depicted in brackets), continuous variables as mean ± SD. Statistical significance was tested using Mann-Whitney test for continuous variables, using Chi-square test for diagnosis and Fisher's exact test for categorical variables. * indicates significance between non-smokers and smokers (*p<0.05, **p<0.01, ***p<0.001). | | |

**Supplementary Table S3: Clinical characteristics of patients with or without arterial hypertension (HTN).**

|  | **no HTN (n=53)** | **HTN (n=253)** |
| --- | --- | --- |
| **BMI (kg/m^2^)** | 25.5 ± 4.3 | 28.2 ± 4.3 *** |
| **Age (years)** | 61.7 ± 9.6 | 65.3 ± 8.2 * |
| **CRP (mg/L)** | 7.5 ± 35.0 | 6.3 ± 17.7 |
| **Creatinine (mg/dL)** | 1.1 ± 1.3 | 1.0 ± 0.3 |
| **Prior MI (%)** | 22.6 (12) | 28.5 (72) |
| **Sex (% male)** | 79.2 (42) | 78.3 (198) |
| **Diabetes Mellitus Type 2 (%)** | 9.4 (5) | 24.9 (63) * |
| **Smoking (%)** | 54.7 (29) | 51.4 (130) |
| **Serum Glucose (mg/dL)** | 101 ± 23 | 121 ± 35 ** |
| **HbA1c (%)** | 5.8 ± 0.4 | 6.3 ± 0.9 ** |
| **Leukocytes (x10^6^/mL)** | 7.7 ± 2.9 | 7.2 ± 2.3 |
| **Total Cholesterol (mg/dL)** | 201 ± 55 | 184 ± 42 |
| **Triglycerides (mg/dL)** | 114 ± 55 | 163 ± 100 ** |
| **LDL (mg/dL)** | 107 ± 46 | 101 ± 34 |
| **VLDL (mg/dL)** | 31.6 ± 14.8 | 35.5 ± 18.3 |
| **HDL (mg/dL)** | 58.5 ± 19.4 | 45.6 ± 13.7 *** |
| **ApoB (µg/mL)** | 216 ± 154 | 203 ± 152 |
| **Hypercholesterolemia (%)** | 22.6 (12) | 48.2 (122) *** |
| **Obesity (%)** | 13.2 (7) | 27.7 (70) * |
| **Metabolic Syndrome (%)** | 5.7 (3) | 58.5 (148) *** |
| **Total IgG (mg/mL)** | 5.0 ± 4.6 | 4.9 ± 4.5 |
| **Total IgM (mg/mL)** | 1.7 ± 1.2 | 1.5 ± 1.1 |
| Categorical variables are expressed as percentages within the groups (total number depicted in brackets), continuous variables as mean ± SD. Statistical significance was tested using Mann-Whitney test for continuous variables, using Chi-square test for diagnosis and Fisher's exact test for categorical variables. * indicates significance between no HTN and HTN (*p<0.05, **p<0.01, ***p<0.001). | | |

**Supplementary Table S4: Clinical characteristics of lean and obese patients.**

|  | **Lean (n=229)** | **Obese (n=78)** |
| --- | --- | --- |
| **BMI (kg/m^2^)** | 25.8 ± 2.6 | 33.4 ± 3.6 *** |
| **Age (years)** | 65.4 ± 8.5 | 62.7 ± 8.5 ** |
| **CRP (mg/L)** | 7.0 ± 24.1 | 5.2 ± 8.4 |
| **Creatinine (mg/dL)** | 1.1 ± 0.7 | 1.0 ± 0.2 |
| **Prior MI (%)** | 28.4 (65) | 24.2 (19) |
| **Sex (% male)** | 76.9 (176) | 83.3 (65) |
| **Diabetes Mellitus Type 2 (%)** | 18.3 (42) | 33.3 (26) ** |
| **Smoking (%)** | 49.3 (113) | 59.0 (46) |
| **Serum Glucose (mg/dL)** | 116 ± 36 | 123 ± 28 * |
| **HbA1c (%)** | 6.1 ± 0.8 | 6.6 ± 1.0 ** |
| **Leukocytes (x10^6^/mL)** | 7.3 ± 2.4 | 7.5 ± 2.4 |
| **Total Cholesterol (mg/dL)** | 187 ± 47.2 | 187 ± 35 |
| **Triglycerides (mg/dL)** | 138 ± 70 | 205 ± 133 *** |
| **LDL (mg/dL)** | 102 ± 37 | 101 ± 31 |
| **VLDL (mg/dL)** | 32.2 ± 14.0 | 41.8 ± 23.8 ** |
| **HDL (mg/dL)** | 49.8 ± 15.6 | 40.8 ± 11.7 *** |
| **ApoB (µg/mL)** | 206 ± 153 | 203 ± 150 |
| **Arterial Hypertension (%)** | 79.9 (183) | 89.7 (70) * |
| **Hypercholesterolemia (%)** | 41.0 (94) | 51.3 (40) |
| **Metabolic Syndrome (%)** | 36.2 (83) | 87.2 (68) *** |
| **Total IgG (mg/mL)** | 4.6 ± 4.4 | 5.7 ± 4.9 * |
| **Total IgM (mg/mL)** | 1.5 ± 1.1 | 1.6 ± 1.3 |
| Categorical variables are expressed as percentages within the groups (total number depicted in brackets), continuous variables as mean ± SD. Statistical significance was tested using Mann-Whitney test for continuous variables, using Chi-square test for diagnosis and Fisher's exact test for categorical variables. * indicates significance between lean and obese (*p<0.05, **p<0.01, ***p<0.001). | | |

**Supplementary Table S5: Clinical characteristics of patients with or without metabolic syndrome (MS).**

|  | **no MS (n=156)** | **MS (n=151)** |
| --- | --- | --- |
| **BMI (kg/m^2^)** | 26.0 ± 3.4 | 29.5 ± 4.6 *** |
| **Age (years)** | 64.8 ± 8.6 | 64.7 ± 8.5 |
| **CRP (mg/L)** | 6.0 ± 22.7 | 6.9 ± 19.8 |
| **Creatinine (mg/dL)** | 1.0 ± 0.7 | 1.1 ± 0.4 * |
| **Prior MI (%)** | 26.9 (42) | 27.8 (42) |
| **Sex (% male)** | 75.6 (118) | 81.5 (123) |
| **Diabetes Mellitus Type 2 (%)** | 6.4 (10) | 38.4 (58) *** |
| **Smoking (%)** | 49.4 (77) | 54.3 (82) |
| **Serum Glucose (mg/dL)** | 103 ± 22 | 129 ± 37 *** |
| **HbA1c (%)** | 5.9 ± 0.5 | 6.5 ± 0.9 *** |
| **Leukocytes (x10^6^/mL)** | 7.3 ± 2.4 | 7.4 ± 2.5 |
| **Total Cholesterol (mg/dL)** | 184 ± 44 | 189 ± 44 |
| **Triglycerides (mg/dL)** | 104 ± 38 | 196 ± 108 *** |
| **LDL (mg/dL)** | 101 ± 37 | 103 ± 35 |
| **VLDL (mg/dL)** | 28.1 ± 12.6 | 40.0 ± 19.5 *** |
| **HDL (mg/dL)** | 53.1 ± 15.8 | 42.9 ± 13.0 *** |
| **ApoB (µg/mL)** | 220 ± 157 | 190 ± 146 * |
| **Arterial Hypertension (%)** | 67.3 (105) | 98.0 (148) *** |
| **Hypercholesterolemia (%)** | 30.1 (47) | 57.6 (87) *** |
| **Obesity (%)** | 6.4 (10) | 45.0 (68) *** |
| **Total IgG (mg/mL)** | 4.8 ± 4.5 | 5.0 ± 4.5 |
| **Total IgM (mg/mL)** | 1.5 ± 1.1 | 1.5 ± 1.2 |
| Categorical variables are expressed as percentages within the groups (total number depicted in brackets), continuous variables as mean ± SD. Statistical significance was tested using Mann-Whitney test for continuous variables, using Chi-square test for diagnosis and Fisher's exact test for categorical variables. * indicates significance between no MS and MS (*p<0.05, **p<0.01, ***p<0.001). | | |

**Supplementary Table S6: Prevalence of CAD or ACS does not associate with total IgG and IgM levels.** Total IgG and IgM levels were quantified in plasma samples cohort using a Flex Set Cytometric Bead Array (BD Biosciences). Patients were grouped by diagnosis (%) among quartiles (Q) of total IgG or IgM levels. CAD, coronary artery disease; ACS, acute coronary syndrome.

|  | **Total IgG** | | | |  | **Total IgM** | | | |
| --- | --- | --- | --- | --- | --- | --- | --- | --- | --- |
|  | **Q1** | **Q2** | **Q3** | **Q4** |  | **Q1** | **Q2** | **Q3** | **Q4** |
| **no CAD** | 23.38 | 9.09 | 20.78 | 34.21 |  | 23.68 | 12.99 | 24.68 | 25.00 |
| **CAD** | 55.84 | 67.53 | 57.14 | 52.63 |  | 59.21 | 61.04 | 48.05 | 65.79 |
| **ACS** | 20.78 | 23.38 | 22.08 | 13.16 |  | 17.11 | 25.97 | 27.27 | 9.21 |

**Supplementary Figures**

**Supplementary Figure S1. Normalized anti-ApoB IgG plasma levels are increased in patients at high cardiovascular risk.** Anti-ApoB IgG plasma levels were quantified by ELISA, normalized to total IgG levels, and grouped according to (A) patient diagnosis or (B-F) cardiometabolic risk factors. CAD, coronary artery disease; ACS, acute coronary syndrome; DM, Diabetes Mellitus; HTN, hypertension; MS, Metabolic Syndrome. Data are presented as median.

**Supplementary Figure S2. Associations of anti-ApoB IgG and IgM plasma levels with plasma CRP levels.** Anti-ApoB (A) IgG and (B) IgM plasma levels were quantified by ELISA and grouped according to patient plasma CRP levels. CRP, C-Reactive Protein. Data are presented as median.

**Supplementary Figure S3. Distribution of anti-ApoB single peptide IgG levels across individual patients.** 10 normotensive, lean patients (“low” CVD risk) and 10 hypertensive, obese patients (“high” CVD risk) were selected from the TRAFIC cohort. Plasma auto-antibodies binding single ApoB-peptides were quantified by ELISA. Anti-ApoB single peptide IgG levels across all 20 patients (A). Individual antibody levels across patients (rows) and single peptides (columns). To display inter-peptide variability, values (RLU-blank) for each patient were normalized as row Z-score (((individual value)-row median value)/row standard deviation). RLU: Relative Luminescence Unit, Blank: background signal. Data are presented as median (A).

**Supplementary Figure S4. Distribution of anti-ApoB single peptide IgG levels across patients with low and high cardiovascular risk.** 10 normotensive, lean patients (“low” CVD risk) and 10 hypertensive, obese patients (“high” CVD risk) were selected from the TRAFIC cohort. Plasma auto-antibodies binding single ApoB-peptides were quantified by ELISA. Anti-ApoB single peptide IgG levels across patients with a low- and high risk for CVD. RLU: Relative Luminescence Unit, Blank: background signal. Data are presented as median ± 95% CI.

**Supplementary Figure S5.** **Associations of normalized anti-ApoB IgG plasma levels with patient lipid profiles.** Anti-ApoB IgG plasma levels were quantifed by ELISA, normalized to total IgG levels, and divided into quartiles (Q) of patient plasma levels of (A) triglycerides, (B) total cholesterol, (C) VLDL, (D) LDL, (E) HDL, and (F) ApoB. VLDL, very low-density lipoprotein; LDL, low density lipoprotein; HDL, high density lipoprotein. Data are presented as median ± 95% CI.

**Supplementary Figure S6.** **Normalized anti-ApoB IgM plasma levels negatively correlate with cardiovascular risk factors.** Anti-ApoB IgM plasma levels were quantified by ELISA, normalized to total IgM levels, and grouped according to (A) patient diagnosis or (B-F) cardiometabolic risk factors. CAD, coronary artery disease; ACS, acute coronary syndrome; DM, Diabetes Mellitus; HTN, hypertension; MS, Metabolic Syndrome. Data are presented as median.

**Supplementary Figure S7.** **Associations of anti-ApoB IgM plasma levels with patient lipid profiles.** Anti-ApoB IgM plasma levels were quantified by ELISA and divided into quartiles (Q) of patient plasma levels of (A) triglycerides, (B) total cholesterol, (C) VLDL, (D) LDL, (E) HDL, and (F) ApoB. VLDL, very low-density lipoprotein; LDL, low density lipoprotein; HDL, high density lipoprotein. Data are presented as median ± 95% CI.

**Supplementary Figure S8.** **Associations of normalized anti-ApoB IgM plasma levels with patient lipid profiles.** Anti-ApoB IgM plasma levels were quantified by ELISA, normalized to total IgM levels, and divided into quartiles (Q) of patient plasma levels of (A) triglycerides, (B) total cholesterol, (C) VLDL, (D) LDL, (E) HDL, and (F) ApoB. VLDL, very low-density lipoprotein; LDL, low density lipoprotein; HDL, high-density lipoprotein. Data are presented as median ± 95% CI.

**Supplementary References**

1. Wolf D, Gerhardt T, Winkels H, Michel NA, Pramod AB, Ghosheh Y, et al. Pathogenic Autoimmunity in Atherosclerosis Evolves From Initially Protective Apolipoprotein B100-Reactive CD4(+) T-Regulatory Cells. Circulation. 2020;142(13):1279-93.
